# From patient to partner: Bridging the divide from laboratory to patient through a “Dragons' Den” style model of co-creation in cancer research^☆^

**DOI:** 10.1016/j.tipsro.2026.100440

**Published:** 2026-10-21

**Authors:** Ashwini Job, Beverly Agass, Charlotte E. Coles, Helene Judge, Andrew Land, Deborah Pitfield, Hilary Stobart, Amy Tang, Rhys Grant, Nicola Le Blond, Neil G. Burnet

**Affiliations:** a Radiation Research Patient and Public Involvement and Engagement Group, University of Cambridge, Early Cancer Institute, Cambridge Biomedical Campus, Cambridge, CB2 0AH, UK; b Department of Oncology, University of Cambridge, Early Cancer Institute, Cambridge Biomedical Campus, Cambridge, CB2 0AH, UK

**Keywords:** Person-focused care, Radiation Therapy, Patient-reported outcomes, Lived experience

## Abstract

Patient and public involvement and engagement (PPIE) in cancer research is increasingly recognised as valuable; yet meaningful participation early in the cancer research pathway remains less common, particularly in pre-clinical science [1]. The Radiation Research Patient and Public Involvement and Engagement (PPIE) group at Cambridge has adapted a format similar to one used in a popular UK TV programme to develop a “Dragons' Den” style model of involvement. In this model, researchers pitch their proposed studies to a panel of patients and carers who provide structured, direct feedback using their lived experience. We describe the delivery model, early impact measures and its potential for broader adoption.

## Highlights

• Patient and Public Involvement and Engagement (PPIE) in cancer research is valuable.
• More meaningful patient participation is required in pre-clinical stages.
• The Cambridge patient group used a “Dragons' Den” style model.
• The patient panel provide structured, direct feedback using their lived experience.
• This model is well-received by patients and researchers alike.

## Introduction

“If we want better outcomes, we must design research together with the people who live its consequences”- quote from patient ‘Dragon’.

Patient and public involvement and engagement (PPIE) in cancer research has evolved considerably over the past two decades. Nevertheless, genuine co-creation, in which patients are active partners from the earliest stages of research design, remains limited. While PPIE is seen commonly in clinical trials, the co-creation gap is more pronounced in pre-clinical and laboratory-based discovery science [1].

Radiotherapy research exemplifies this gap. The field encompasses highly technical science, complex clinical-translational work, and direct patient care. Every experiment in radiobiology and every treatment technique development should ultimately have line of sight to a person receiving radiotherapy. Yet the realities of treatment burden cannot be derived from laboratory data alone. This is why early patient input is paramount, because without it opportunities to shape research in meaningful and patient-centric ways can be missed, precisely at the point where it could have the greatest impact.

In Cambridge we developed a structured, scalable PPIE model that draws on the format of the BBC television programme “Dragons' Den” [2]. In this approach, researchers present their early project proposals to a panel of patients and carers, who offer direct and candid feedback based on lived experience. The model was designed to be practical, replicable, and genuinely empowering for both researchers and patients. Here we report on its development, delivery, and early impact data.

## Materials and methods

### Setting and participants

The Radiation Research PPIE Group supports radiation research across the University of Cambridge and Cambridge University Hospitals NHS Foundation Trust and partners with Cancer Research UK RadNet Cambridge [3]. The group comprises a diverse set of patients with personal experience of radiotherapy for a range of cancer diagnoses (Table 1), as well as radiation researchers.

**Table 1:** Diversity Characteristics of the Dragons' Den PPIE panel. PPIE, Patient and Public Involvement and Engagement.

Diversity Characteristics	Category	Number
Gender	Male	1
	Female	3
Age Range	30–40	1
	40–80	3
Ethnicity	Asian	1
	White	3
Role	Patient	4
	Carer	0

The “Dragons' Den” style model was developed collaboratively within the group and adapted from the well-known television format. In the TV show, entrepreneurs present business proposals to a panel who have the expertise to offer challenging, constructive feedback. The core difference with our model is one of expertise: lived experience of cancer and its treatment is positioned as the primary expertise the panel brings.

Researchers, including scientists who had never previously engaged directly with patients in a research or clinical context, were given a short time to present their project - its aims, methodology, and relevance to patients, without assuming specialist knowledge in their audience. The panel then entered into open dialogue with the researcher, including discussing the objectives of the research and challenging assumptions about what patients would and would not find acceptable in the proposed research study.

### Event structure

The model was delivered across three events (Table 2). The first “Dragons' Den” session (May 2025), invited four research teams to present proposals spanning discovery radiation science through to clinical-translational work. Each presentation was followed by a discussion in which the PPIE panel offered feedback on study design, patient-facing communication, and the practical realities of treatment burden. A second event (September 2025) repeated the format with a second cohort of five research teams, one presenting for a second time.

**Table 2:** Three-stage “Dragons' Den” delivery model. PPIE, Patient and Public Involvement and Engagement.

Event	Date	Cohort	Focus
1st “Dragons' Den”	May 2025	4 research teams	Initial pitches: discovery science to clinical-translational work; open PPIE feedback
2nd “Dragons' Den”	September 2025	5 research teams (2nd cohort)	Repeat process; 4 new researchers experiencing the panel's involvement for the first time and 1 researcher presenting an updated proposal
Review of impact event	January 2026	8 returning researchers	Researchers reported back on the key question: what changed in the research plan as a result of PPIE involvement? A physical- virtual hybrid model was used for this event due to attendee location.

A third event, (January 2026), brought all of the original participating researchers back together to report on how their work had changed as a result of the panel's involvement. This impact review was integral to the model design: it closed the feedback loop, held researchers accountable, and generated the outcome data reported here. Researchers and PPIE members also provided feedback on the usefulness of the model.

## Results and discussion

### Quantitative impact

At the wrap-up event, researchers rated the helpfulness of the “Dragons' Den” style format on a ten-point scale and indicated whether they would recommend the model to colleagues. Researchers also reported whether, and in what domains, their work had changed as a result of PPIE. PPIE panel members provided separate feedback on their experience.

Data from the January 2026 impact review event are summarised below and in Fig. 1. Six of eight participating research teams (75%) reported direct changes to their study design as a result of PPIE feedback. These were not marginal adjustments: researchers described changes to protocol design, revisions to patient-facing communication materials, and amendments to grant applications. The mean researcher rating of this approach was 8.9 out of 10 for helpfulness (Fig. 2). All eight researchers (100%) stated they would recommend the model to colleagues.

**Fig. 1:**
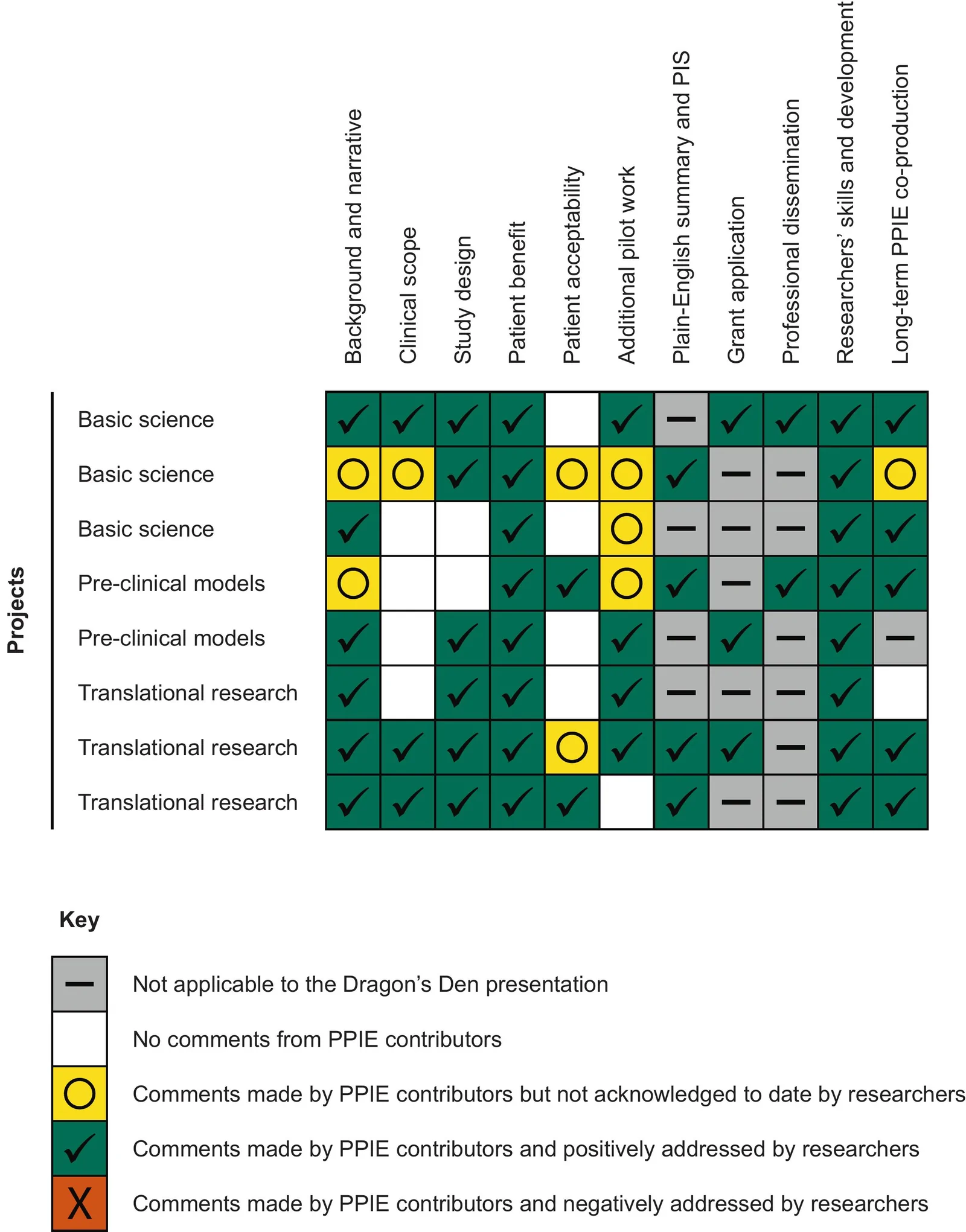
Colour-coded matrix showing the extent to which patient and public contributor feedback in the “Dragons' Den” sessions was acknowledged and acted upon across the project domains. PPIE, Patient and Public Involvement and Engagement; PIS, Patient Information Sheet.

**Fig. 2:**
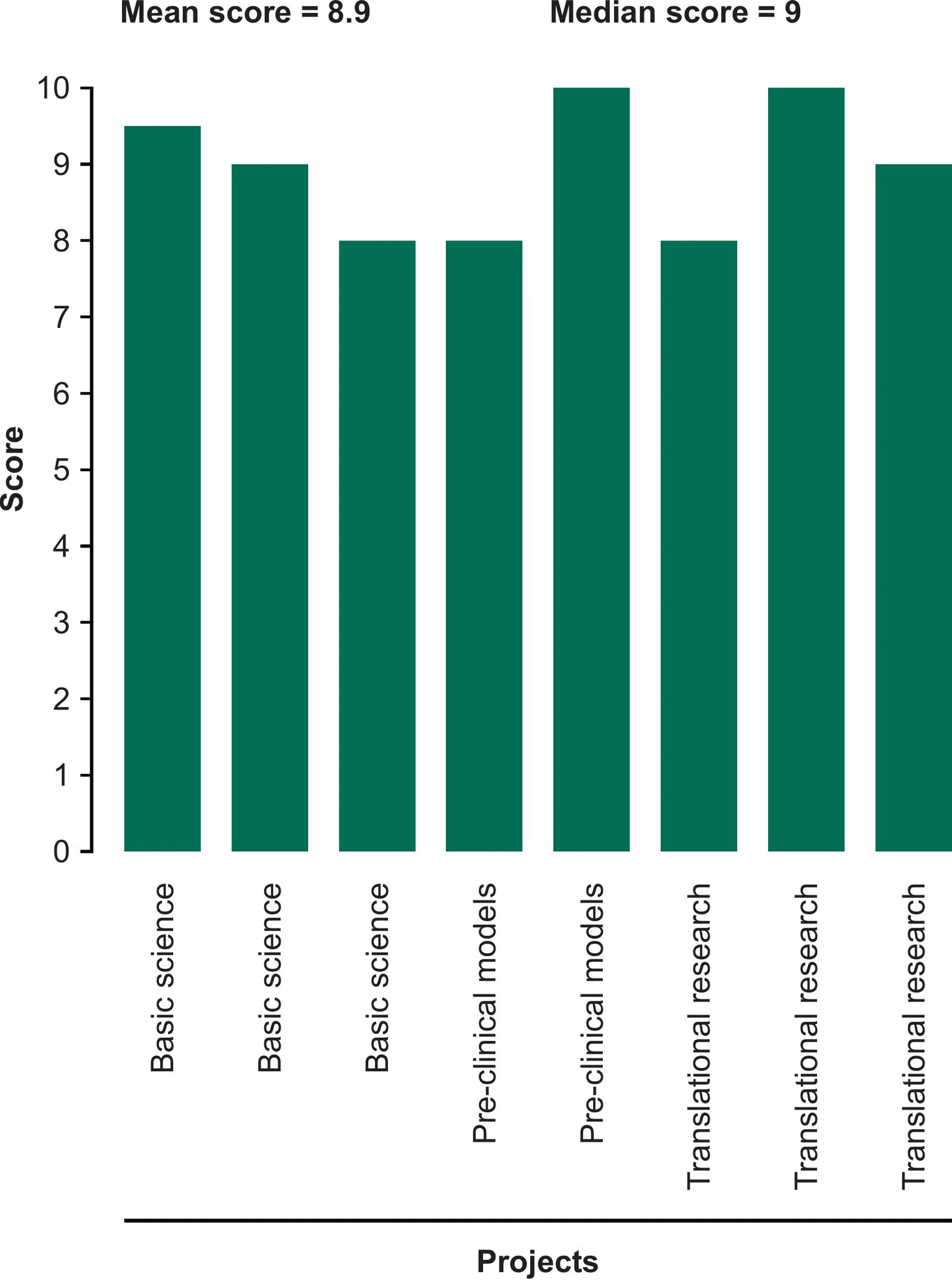
Researcher scores when asked how helpful the “Dragons' Den” format was on a scale of 1–10. The mean score, represented by the dashed line, was 8.9. The median score was 9, with a score-range from 8 to 10.

Notably, five of the eight projects that participated in the “Dragons' Den” sessions were pre-clinical radiation studies, a domain in which meaningful PPIE involvement has historically been difficult to achieve [1]. The finding that the Radiation Research PPIE Group was judged to have contributed substantially through our “Dragons' Den” method is therefore particularly important.

### Qualitative and cultural impact

One example of a study design which changed as a result of the PPIE feedback is where a researcher, following feedback from the Dragons' Den, held a focus group with the PPIE panel to obtain feedback. The insights from the Dragons' Den session confirmed the need for thorough clinical sample interrogation, leading to a funded discovery project focussed on biomarkers, using a novel platform. The feedback from the focus group also steered future plans toward more convenient sampling methods already integrated into the patient's care plan (e.g. sampling to be scheduled at the same time as pre-scheduled patient visits). The feedback also revealed the need to address side effects of the proposed treatment on natural aging and healthy tissue, challenging the former more narrow disease-focussed approach.

When queried, qualitative feedback from researchers described this Dragon's Den format as providing a “supportive but positively challenging environment” and “an excellent way of getting PPIE involvement early in study design”. It was described as having “outperformed expectations.” For several pre-clinical researchers, this represented their first substantive direct encounter with patients in a research context.

These responses suggest a cultural as well as a practical shift: the “Dragons' Den” style model appears to reduce the perceived barrier between laboratory-based scientists and the patient community.

From the perspective of PPIE panel members, when asked for feedback following the event, it was reported that the model generated genuine feelings of being valued and as equals rather than consulted as a formality. Members described feeling inspired by the science and engaged by the opportunity to contribute meaningfully to research at the design stage. The exposure to developing research ideas also provided a sense of optimism for future developments in cancer care. This reciprocal experience, in which both researchers and patient partners report changes in how they perceive one another's contribution, may be among the most significant impacts of the model.

### Why the “Dragons' Den” style format works

Several features of this approach contribute to its effectiveness. First, the format creates a shared space in which two distinct forms of knowledge – scientific expertise and lived experience of illness - are brought into genuine dialogue at the earliest opportunity. Rather than positioning PPIE as a requirement to be satisfied, the “Dragons' Den” framing makes it natural for researchers to ask questions, seek clarification, and leave with a richer understanding of what their work means for the people it is ultimately intended to help.

Second, the format is structured and time-bounded. Unlike open consultation exercises, the model has a clear sequence: presentation, open questioning, and written follow-up. This structure makes it practical for busy researchers and ensures that PPIE input is directed at specific aspects of study design.

Third, the follow-up impact review event is an important feature. By requiring researchers to return and report on what changed, the model reinforces accountability and makes the co-creation loop visible. This mechanism differs from many conventional PPIE frameworks as feedback and subsequent action is recorded after the follow-up discussion between patients and researchers. This dialogue can be mutually rewarding as evidenced by the comments captured from both parties.

### Limitations

As an early-stage evaluation, this work reflects experience from a single centre and a modest cohort, whereas future work would benefit from adopting the model with larger numbers and potentially across sites. Researchers and PPIE panel-members were voluntary, which is largely typical of early PPIE initiatives. We acknowledge that while the researchers involved in the study reported positive outcomes, not all researchers in the wider community maybe as willing to engage. However, the encouraging outcomes reported here would also be usefully complemented by independent evaluation and longer-term follow-up, to build on this promising initial evidence and further strengthen the case for wider adoption of the model.

### Future considerations

Looking ahead, we aim to build on this early evidence by increasing the numbers of both PPIE and researchers, using an established quantitative framework for evaluation and to develop a standardised toolkit that would enable other centres to adopt the model to their own settings. The standardised toolkit would also help the model scale across centres and potentially across countries. The model also enables demonstrating to early career researchers the value of embedding PPIE input from the outset.

### Implications for radiotherapy research

The radiotherapy field presents specific opportunities and challenges for PPIE. Scientific complexity and the pre-clinical nature of much radiobiology research can make patient involvement feel remote and disconnected from the science. Simultaneously, the treatment experience in radiotherapy, involving multiple hospital visits, side effects due to other treatments, and potential toxicity, generates forms of knowledge that are directly relevant to research design. Embedding patient voices early can influence research questions to ensure these are important to patients and inform trial design to include patient-centric endpoints and minimise patient burden. It can also help pre-clinical researchers appreciate how to make their work relevant to patients, providing a line of sight to patient benefit even when the science is still several steps removed from clinical implementation.

As a result, these positive “Dragons' Den” interactions may potentially facilitate strong, competitive grant applications with improved chance of success of funding, research delivery and ultimately better patient outcomes and experiences [4]. In addition, these early-stage, patient-researcher partnerships will facilitate public trust in, and understanding of, cancer discovery science and clinical-translational research.

## Conclusion

The “Dragons' Den” style PPIE model demonstrates that structured, high-impact patient involvement is not only achievable and measurable at the earliest stages of radiotherapy research, but that it is also valued by researchers and patients alike. The model is time-efficient, practically deliverable, and can generate measurable changes in research design. It also creates a positive shift in the relationship between researchers and patients that appears to have value beyond any single study. By treating lived experience as genuine expertise, and by building in accountability through a structured impact review, this approach offers a transferable pathway to genuine co-creation in cancer research.

## CRediT authorship contribution statement

**Ashwini Job:** Writing – review & editing, Writing – original draft. **Beverly Agass:** Writing – review & editing, Writing – original draft. **Charlotte E. Coles:** Writing – review & editing. **Helene Judge:** Writing – review & editing. **Andrew Land:** Writing – review & editing. **Deborah Pitfield:** Writing – review & editing. **Hilary Stobart:** Writing – review & editing. **Amy Tang:** Writing – review & editing. **Rhys Grant:** Writing – review & editing. **Nicola Le Blond:** Writing – review & editing, Project administration. **Neil G. Burnet:** Writing – review & editing, Writing – original draft.

## Declaration of competing interest

The authors declare that they have no known competing financial interests or personal relationships that could have appeared to influence the work reported in this paper.
